# Supplementary material for: Changes in Body Mass, Physical Activity, and Dietary Intake during the COVID-19 Pandemic Lockdowns in Canadian University Students
Source: Biology (Basel). 2023 Feb 17;12(2):326. doi: 10.3390/biology12020326 (PMC9953033; doi:10.3390/biology12020326)

Appendix A: The survey

Q1.

Project Title: Impacts of the COVID-19 Pandemic on Eating Habits and Behaviours in Canada Among Post-Secondary Students

Supervising Investigator: Dr. Nota Klentrou, Professor
Department of Kinesiology
Brock University
Email: nklentrou@brocku.ca

INVITATION
You are invited to participate in a research study to explore how the COVID-19 pandemic has impacted eating habits and behaviour patterns before, during and after the start of stay-at-home, social distancing strategies and closure of outdoor recreation areas in mid-March, 2020 in Canada. It also explores the impact of the closure of outdoor recreation areas on your perceptions of your mental, physical and overall health.

ELIGIBILITY
To be eligible to participate in this study you must:

- Attended a Canadian University for undergraduate studies during the 2019/2020 school year,
- Be a minimum age of 18 years old, and;
- Be residing within Canada for the COVID-19 pandemic,

WHAT'S INVOLVED
As a participant, you will be asked to complete an online survey. Participation will take approximately 30-60 minutes of your time.

POTENTIAL BENEFITS AND RISKS
You may benefit from this study by having the chance to reflect on your eating habits and behaviors, outdoor recreation and mental health practices, and contemplate the importance of these factors in your life and overall wellbeing. You may also learn about nutrition, managing stress and outdoor recreation opportunities by considering the scope and range of resources available in Canada. Finally, aggregated findings from this study will be shared with government agencies, land managers, and private service providers in hopes of informing future decisions about the importance of nutrition and dietary habits and behaviour during restriction and public closures. This will benefit both decision-makers and participants. Findings will also inform the scholarly understanding of dietary habits and behaviour, and outdoor recreation and how access (or lack of access) to public and outdoor recreation areas impacts people.

There may also be risks associated with participation. You may feel some emotional stress when reflecting on loss of access to outdoor recreation and public areas, or COVID-19 circumstances. If you feel undue stress, you should contact your local or provincial mental health service provider or telehealth agency for assistance.

CONFIDENTIALITY

All information you provide is considered confidential; your name will not be included or, in any other way, associated with the data collected in the study. Furthermore, because our interest is in the average responses of the entire group of participants, you will not be identified individually in any way in written reports of this research. To protect your privacy, we recommend you complete the survey in a private location and clear your browser cache after submitting the survey. Data collected during this study will be electronically stored indefinitely on the researchers' computers in a password protected file as it may be necessary to allow the data to be used for future comparative purposes by our group or by colleagues, students, or other researchers. After the completion of the study, hardcopy files will be stored for 5 years, after which all of the hardcopy data will be deleted.

VOLUNTARY PARTICIPATION
Participation in this study is voluntary. If you wish, you may decline to answer any questions or participate in any component of the study. Further, you may decide to withdraw from this study at any time and may do so without any penalty or loss of benefits to which you are entitled. If you choose to withdraw, simply close your browser window and do not return to the survey website. If you choose to withdraw and do not want any data you've provided included in the study, you can email Madison Bell with the date, approximate time, and approximate question at which you stopped the survey. This will compromise anonymity, but allow the researchers to find your file and delete it from the database.

PUBLICATION OF RESULTS
Results of this study may be published in professional journals, presented at conferences, and shared with governmental agencies. Feedback about this study will be available upon completion. You may request an executive summary of the research at the end of the survey by entering an email address where indicated. This will not be connected to your survey responses and only be used to send the executive summary to you.

CONTACT INFORMATION AND ETHICS CLEARANCE
If you have any questions about this study or require further information, please contact Madison Bell, Principal Student Investigator, using the contact information provided above. This study has been reviewed and received ethics clearance through the Research Ethics Board at Brock University [File #____]. If you have any comments or concerns about your rights as a research participant, please contact the Research Ethics Office at (905) 688-5550 Ext. 3035

Thank you for your assistance in this project. You may download a copy of this form here (Consent form.pdf) if you would like one for your records.
Please **Select** the appropriate button to indicate whether you would or would not wish to participate in this questionnaire. If you select the "I do not agree" to participate, this survey will close.

- (●) I AGREE to participate in this study and complete the survey. I confirm I am an undergraduate student enrolled at a Canadian post-secondary University for the 2019/20 school year, that I am 18 years of age or older, and am residing in Canada during the COVID-19 pandemic. I understand that I do not have to answer any question that I am uncomfortable answering and that I may end my participation at any time. I understand my responses are anonymous, confidential and may be stored for later research. Selecting this button and clicking the arrow at the bottom of the page will start the survey.
- ( ) I DO NOT AGREE to participate in this study and complete the survey at this time. I am aware that I can return to this link and complete it at another time. Selecting this button and clicking the arrow at the bottom of the page will close the survey.

Q2. This survey is best completed on a laptop or computer, as some questions have a wider format. If you are on a mobile device you will be able to continue with the survey, it may just take you a few minutes longer to complete.

Specification of terminology:

*Prior to/before the COVID-19 pandemic, will stand for meaning the time period before the World Health Organization declared a global pandemic and before Canada began experiencing restrictions due to the pandemic, therefore any time before March 13th of 2020.

*During the COVID-19 pandemic, will stand for meaning the time period after the World Health Organization declared a global pandemic and Canada began experiencing restrictions due to the pandemic, therefore any time after March 13th of 2020.

Q3. This survey should take you approximately 30-45 minutes to complete.

A reminder that all answers are anonymous and confidential.

There are three sections:
1. Background: demographic, physical activity and stress level questions
2. Eating Behaviours: questions regarding reasons for eating/not eating
3. Food Frequency Questionnaire: a valid and reliable questionnaire for estimating food intake and nutrition profiles

Q4.
SECTION 1: The following questions are to provide us with some background on your demographic, your current living situation and lifestyle, as well as looking to compare with prior to COVID-19 (before restrictions). Please answer each question with the option that best represents you.

Q5. What age bracket do you fall into as of today?

- ○ 18-24
- ○ 25-39
- ○ 40-59
- ○ 60+

Q6. What is your sex?

Male	Female	Intersex
○	○	○

Q7. What is your race/ethnicity?

[▼]

Q8. Estimated height in feet and inches? (e.g. 5'7")

Q9. Estimated weight in pounds (lbs)?

Q10. Year of study for the 2019/2020 school year?

- Year 1
- Year 2
- Year 3
- Year 4
- Year 5+

Q11.
PRIOR to the COVID-19 pandemic, wherein Canada did you reside while attending school?

- Ontario
- Quebec
- Prairies (Saskatchewan or Manitoba)
- West Coast (B.C. or Alberta)
- East Coast (Nova Scotia, New Brunswick, P.E.I., Newfoundland and Labrador)
- Territories (Northwest, Nunavut, Yukon)
- Did not live in Canada Prior to COVID-19

Q12.

DURING the COVID-19 pandemic, wherein Canada do you reside currently?

- ○ Ontario
- ○ Quebec
- ○ Prairies (Saskatchewan or Manitoba)
- ○ West Coast (B.C. or Alberta)
- ○ East Coast (Nova Scotia, New Brunswick, P.E.I., Newfoundland and Labrador)
- ○ Territories (Northwest, Nunavut, Yukon)

Q13. Please select which best describes your situation. (select all that apply)

	PRIOR to COVID-19 (before March 2020)	DURING COVID-19 (March 2020-today)
Living alone	○	○
Living with family or a significant other	○	○
Living with friends or roommates	○	○
Living in an apartment/condo/residence	○	○
Living in a house/duplex	○	○
Have access to a yard/backyard	○	○
Have access to a gym	○	○
Have access to a pool/lake	○	○
Employed	○	○

Q14.
Please indicate if you have ever been diagnosed with/experience any of the following conditions on a regular basis (select all that apply).

- ☐ High Cholesterol
- ☐ High blood pressure
- ☐ Depression
- ☐ Anxiety disorder
- ☐ Bulimia
- ☐ Anorexia
- ☐ Arthritis
- ☐ Osteoporosis/Osteopenia
- ☐ Broken bone
- ☐ Crohns/Ulcerative Colitis
- ☐ Kidney problems
- ☐ Asthma

- ☐ Chest pain or shortness of breath
- ☐ Diabetes
- ☐ Irritable Bowel Syndrome
- ☐ Cancer
- ☐ Ulcer
- ☐ Food allergies
- ☐ Thyroid conditions
- ☐ Other allergies
- ☐ Celiac disease

Q15.
PRIOR to COVID-19 restrictions, how often did you use/consume any of the following (select all that apply):

	Never	Rarely	Sometimes	Fairly Often	Very Often
Marijuana	○	○	○	○	○
Other Recreational Drugs	○	○	○	○	○
Cigarettes	○	○	○	○	○
Vapes/E-Cigarettes	○	○	○	○	○
Alcohol	○	○	○	○	○

Q16.
DURING the COVID-19 restrictions, how often did you use/consume any of the following (select all that apply):

	Never	Rarely	Sometimes	Fairly Often	Very Often
Marijuana	○	○	○	○	○
Other Recreational Drugs	○	○	○	○	○
Cigarettes	○	○	○	○	○
Vapes/E-Cigarettes	○	○	○	○	○
Alcohol	○	○	○	○	○

Q17.
Over the past 3 months have you noticed any of the following within yourself? (select all that apply).

- ☐ Minor weight gain (0-10 pounds)
- ☐ Minor weight loss (0-10 pounds)
- ☐ Major weight gain (10+ pounds)
- ☐ Major weight loss (10+ pounds)

- [ ] Increased appetite
- [ ] Decreased appetite
- [ ] No change in appetite
- [ ] No change in weight

Q18.
Comparing **before COVID-19** to **during COVID-19**; answer based on the change in how often you participate in the following:

	Less than before	Same as before	More than before
Walking the dog or going for a walk	◯	◯	◯
Light/moderate running or jogging	◯	◯	◯
Moderate/high intensity running	◯	◯	◯
Biking	◯	◯	◯
Swimming	◯	◯	◯
Weight Lifting/ Resistance Training	◯	◯	◯
“At-home” workouts, Yoga, Pilates, etc.	◯	◯	◯
Recreational Sports (volleyball, soccer, tennis, etc.)	◯	◯	◯
Sport Specific training(s) (drills, strength and conditioning, sport practices, etc.)	◯	◯	◯

Q19. **Based on the last 3 months** rank the following from MOST stressful at the top, to LEAST stressful at the bottom. (click, hold and drag to move).

Financial stress (e.g. loss of employment, lack of income, paying bills, affording rent/tuition, etc.)

Social stress (e.g. social media, social distancing restrictions, lack of socialization, restrictions of public areas such as parks, beaches, restaurants, gyms, beauty salons, etc.)

Emotional stress (e.g. mental health, family and or friends afflicted by COVID-19)

Physical Stress (e.g. diagnosed with COVID-19 yourself, lack of physical activity, extreme weight changes, overall health, healthcare restrictions such as medical appointments, therapies or surgeries, etc.)

Workplace stress: (e.g. being a frontline/essential worker, working from home/career changes/ business issues due to COVID-19, etc.)

Stress due to change (e.g. school moved online, lack of available resources, children out of school/ finding childcare, transportation restriction, change of living environment/living arrangements such as moving in with parents, roommates, etc.)

Q20.

SECTION 2: The following questions are to provide us with some background on your current eating behaviours and habits. Please answer each question with the option that best represents you as of today.

Q21. Eating Behaviours

	Never	Rarely	Sometimes	Often	Always
Do you try to eat less than you would like to eat?	○	○	○	○	○
How often do you refuse food or drink offered because you are concerned about your weight?	○	○	○	○	○
Do you watch exactly what you eat?	○	○	○	○	○
Do you deliberately eat foods that are slimming?	○	○	○	○	○
Do you deliberately eat less in order not to become heavier?	○	○	○	○	○
How often do you eat to put on weight?	○	○	○	○	○
How often do you use supplements to put on weight?	○	○	○	○	○
Do the restrictions put in place during the pandemic influence your eating habits?	○	○	○	○	○

Q22. SECTION 3: The following series of questions are about foods you usually eat.

Think about your intake since the start of the COVID-19 restrictions (Mid March 2020) to today. This includes all meals or snacks, at home, in restaurants or take-out. You will be asked specifics such as how often, followed by how much.

*If you don't eat a food (select 'never'), and the following question will ask how much you consume of it, leave that question blank and move on to the next one.

A portion size chart will be given at the beginning of each page to help you visualize different amounts in bowls and or plates.
A=1/4 Cup of Food
B=1/2 Cup of Food
C=1 Cup of Food
D=2 Cups of Food

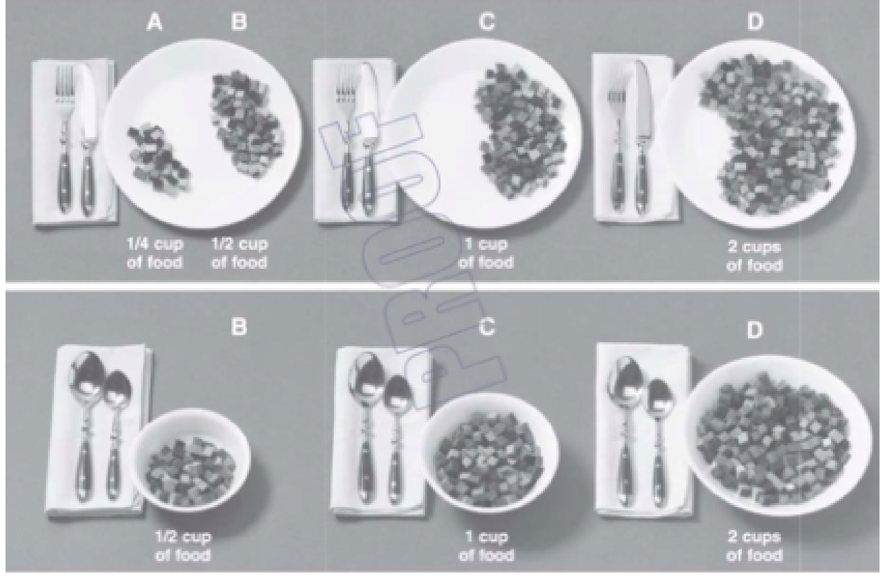

Q24. EGGS and DAIRY FOODS

	How Often?									How much on those days? (see portion size pictures for A-B-C-D)			
	Never	A Few Times per 4 Months	Once per Year	2-3 Times per Month	Once per Week	2 Times per week	3-4 Times per Week	5-6 Times per Week	Every Day	A	B	C	D
Yogurt (not frozen)	○	○	○	○	○	○	○	○	○	○	○	○	○
Cottage Cheese, ricotta cheese	○	○	○	○	○	○	○	○	○	○	○	○	○

Q25. EGGS and DAIRY FOODS

	How Often?									How many on those days?			
	Never	A Few Times per 4 Months	Once per Year	2-3 Times per Month	Once per Week	2 Times per week	3-4 Times per Week	5-6 Times per Week	Every Day	1	2	3	4
Breakfast sandwiches or breakfast burritos with egg or meat	○	○	○	○	○	○	○	○	○	○	○	○	○
Other eggs like scrambled or boiled, or quiche (not egg substitutes)	○	○	○	○	○	○	○	○	○	○	○	○	○
Cream cheese, sour cream, dips (tbsp)	○	○	○	○	○	○	○	○	○	○	○	○	○
Cheese/cheese slices, cheese spread, including in sandwiches and quesadillas	○	○	○	○	○	○	○	○	○	○	○	○	○

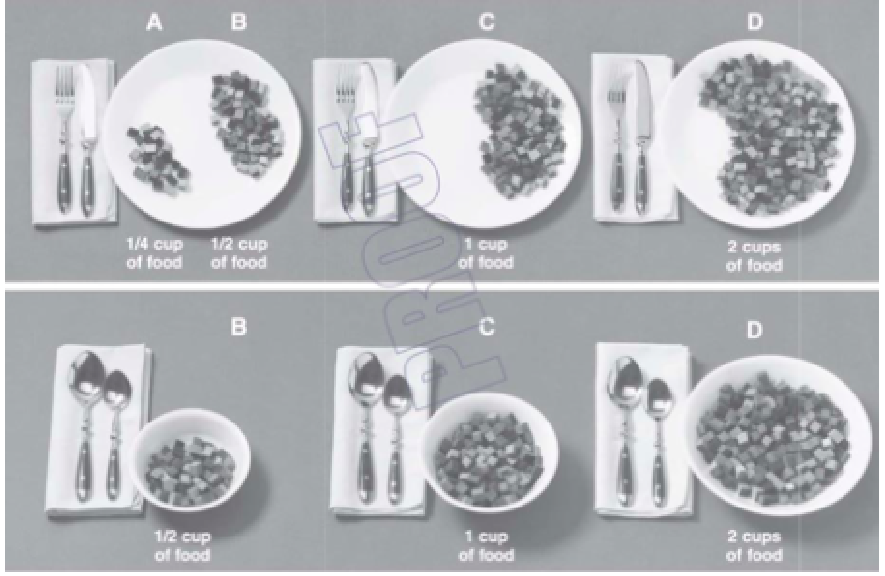

Q27. CEREALS, GRAINS, BREADS

	How Often?									How much on those days? (see portion size pictures for A-B-C-D)			
	Never	A Few Times per 4 Months	Once per Year	2-3 Times per Month	Once per Week	2 Times per week	3-4 Times per Week	5-6 Times per Week	Every Day	A	B	C	D
Cold cereals (ANY KIND, like corn flakes, fibre cereals, sweetened cereals)													
Oatmeals, or whole grain cereal like Wheatena or Ralston													
Click to write Statement 3Grits, cream of wheat, cornmeal mush													
	Never	A Few Times per 4 Months	Once per Year	2-3 Times per Month	Once per Week	2 Times per week	3-4 Times per Week	5-6 Times per Week	Every Day	A	B	C	D
Milk or milk substitutes on cereal													
Brown rice, or dished made with brown rice													
White rice, or dishes made with rice, like rice and beans													

Q28. CEREALS, GRAINS, BREADS

	How Often?									How many on those days?				
	Never	A Few Times per 4 Months	Once per Year	2-3 Times per Month	Once per Week	2 Times per week	3-4 Times per Week	5-6 Times per Week	Every Day	1/2	1	2	3	4

Pancakes, waffles, French toast, crepes														
Breakfast pastries, like muffins, scones, sweet rolls, Danish, Pop Tarts, pan dulce														
Biscuits, NOT counting breakfast sandwiches														
Corn bread, corn muffins, hush puppies														
	Never	A Few Times per 4 Months	Once per Year	2-3 Times per Month	Once per Week	2 Times per week	3-4 Times per Week	5-6 Times per Week	Every Day	1/2	1	2	3	4
Hamburger buns, hotdog buns, submarine or hoagie buns														
Bagels or English muffins, dinner rolls, pita, naan														
Tortillas (not counting in tacos or burritos)														
Any other bread or toast, including white, dark, whole wheat, and what you have in sandwiches														

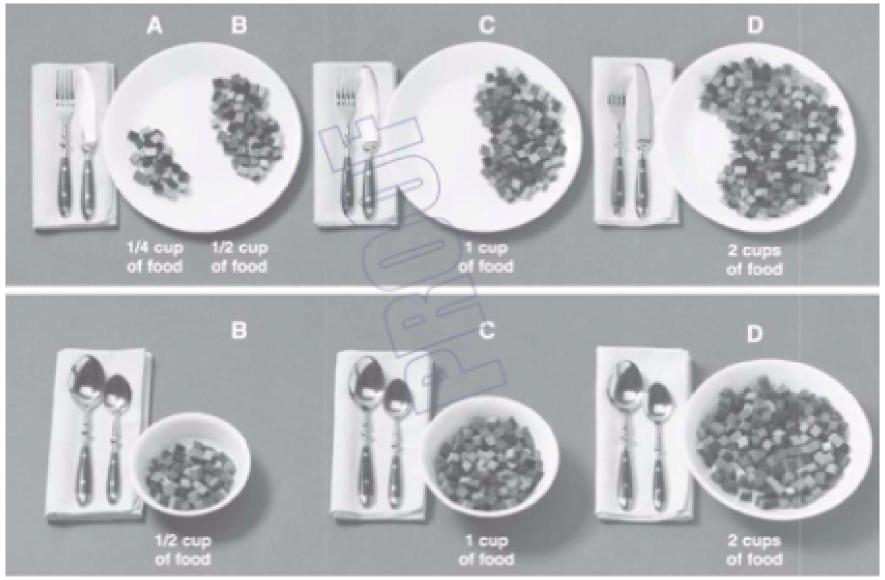

Q30. VEGETABLES

	How Often?									How much on those days? (see portion size pictures for A-B-C-D)			
	Never	A Few Times per 4 Months	Once per Year	2-3 Times per Month	Once per Week	2 Times per week	3-4 Times per Week	5-6 Times per Week	Every Day	A	B	C	D
Broccoli, Chinese broccoli, or Brussels sprouts													
Carrots and mixed vegetables containing carrots													
Corn													
Green beans, string beans, green peas													

	Never	A Few Times per 4 Months	Once per Year	2-3 Times per Month	Once per Week	2 Times per week	3-4 Times per Week	5-6 Times per Week	Every Day	A	B	C	D
Cooked greens like spinach, collards, turnip greens, kale, mustard greens	○	○	○	○	○	○	○	○	○	○	○	○	○
Cabbage, coleslaw, Chinese cabbage	○	○	○	○	○	○	○	○	○	○	○	○	○
Sweet potatoes, yams	○	○	○	○	○	○	○	○	○	○	○	○	○
French fries, home fries, hash browns, tater tots	○	○	○	○	○	○	○	○	○	○	○	○	○
Potatoes (NOT fried), like baked, boiled, mashed, or in stew or potato salad	○	○	○	○	○	○	○	○	○	○	○	○	○
Any other vegetables, like squash, cauliflower, peppers, okra, nopales	○	○	○	○	○	○	○	○	○	○	○	○	○

Q31. VEGETABLES

	How Often?									How many CUPS on those days?				
	Never	A Few Times per 4 Months	Once per Year	2-3 Times per Month	Once per Week	2 Times per week	3-4 Times per Week	5-6 Times per Week	Every Day	1/2	1	2	3	4
Green salad with lettuce or raw spinach	○	○	○	○	○	○	○	○	○	○	○	○	○	○

Q32. VEGETABLES

	How Often?									How much on those days?			
	Never	A Few Times per 4 Months	Once per Year	2-3 Times per Month	Once per Week	2 Times per week	3-4 Times per Week	5-6 Times per Week	Every Day	1/4	1/2	1	2
Raw tomatoes	○	○	○	○	○	○	○	○	○	○	○	○	○

Q33. Vegetables

	How Often?									How many tablespoons on those days?			
	Never	A Few Times per 4 Months	Once per Year	2-3 Times per Month	Once per Week	2 Times per week	3-4 Times per Week	5-6 Times per Week	Every Day	1	2	3	4
Salad dressing	○	○	○	○	○	○	○	○	○	○	○	○	○
Avocado, guacamole	○	○	○	○	○	○	○	○	○	○	○	○	○

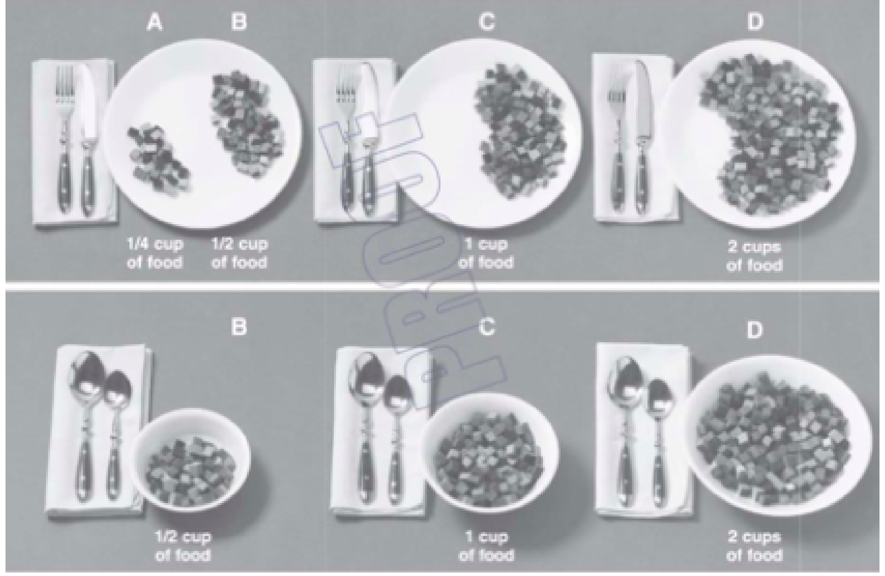

Q35. FRUITS, FROZEN FRUITS

	How Often?									How much on those days? (see portion size pictures for A-B-C-D)			
	Never	A Few Times per 4 Months	Once per Year	2-3 Times per Month	Once per Week	2 Times per week	3-4 Times per Week	5-6 Times per Week	Every Day	A	B	C	D
Watermelon, cantaloupe, honeydew, other melons													
Strawberries or other berries													
Any other fresh fruit, like grapes, plums, mango, fruit salad													
Raisins, dates, other dried fruit													
Canned fruit, like applesauce, fruit cocktail, canned peaches or pineapple													

Q36. FRUITS, FROZEN FRUITS

	How Often?									How many on those days?		
	Never	A Few Times per 4 Months	Once per Year	2-3 Times per Month	Once per Week	2 Times per week	3-4 Times per Week	5-6 Times per Week	Every Day	1/2	1	2
Bananas												
Apples or pears												
Oranges, tangerines, grapefruit												
Peaches and nectarines												

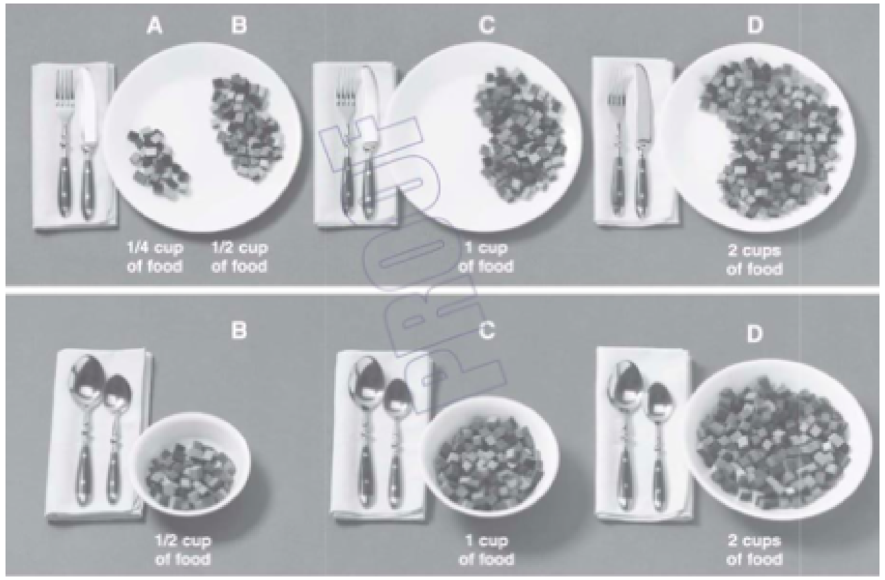

Q38. BEANS, TOFU, and MEAT SUBSTITUTES

	How Often?									How much on those days? (see portion size pictures for A-B-C-D)			
	Never	A Few Times per 4 Months	Once per Year	2-3 Times per Month	Once per Week	2 Times per week	3-4 Times per Week	5-6 Times per Week	Every Day	A	B (1 patty or dog)	C	D
Refried beans, bean burritos, or hummus													
Pinto beans, black beans, kidney beans, baked beans, lentils													
	Never	A Few Times per 4 Months	Once per Year	2-3 Times per Month	Once per Week	2 Times per week	3-4 Times per Week	5-6 Times per Week	Every Day	A	B (1 patty or dog)	C	D
Tofu or lempheh													
Meat substitutes, like veggie burgers, veggie chicken, vegetarian hotdogs or vegetarian lunch meats													

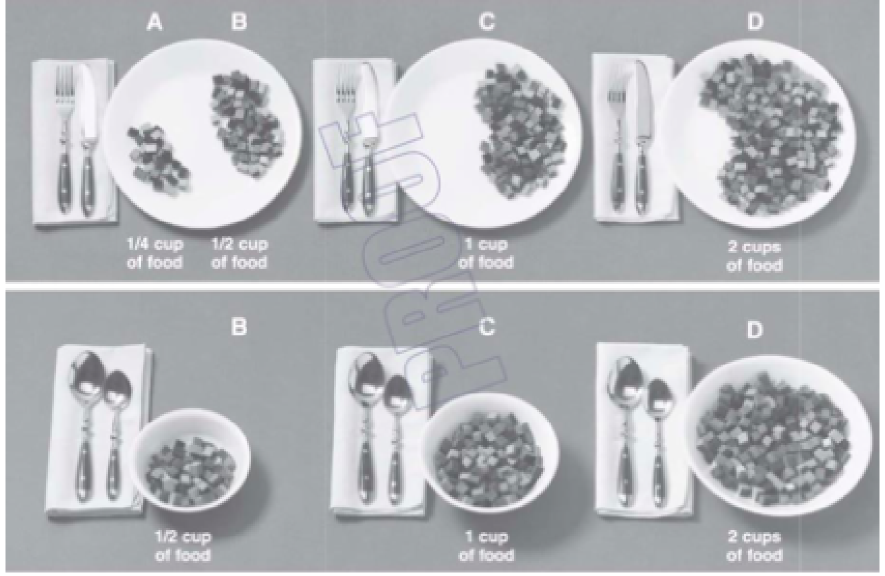

Q40. SOUPS, MIXED DISHES, and NOODLES

	How Often?									How much on those days? (see portion size pictures for A-B-C-D)		
	Never	A Few Times per 4 Months	Once per Year	2-3 Times per Month	Once per Week	2 Times per week	3-4 Times per Week	5-6 Times per Week	Every Day	B	C	D
Split pea, bean, or lentil soup												
Vegetable soup, vegetable beef soup, or tomato soup												
Any other soup, including chicken noodle, cream soups, Cup-A-Soup, ramen												
	Never	A Few Times per 4 Months	Once per Year	2-3 Times per Month	Once per Week	2 Times per week	3-4 Times per Week	5-6 Times per Week	Every Day	B	C	D
Macaroni and cheese												
Spaghetti, lasagna, other pasta with tomato sauce												
Other noodles like plain pasta, pasta salad, soap seca												

Q41. SOUPS, MIXED DISHES, and NOODLES

	How Often?									How many on those days?			
	Never	A Few Times per 4 Months	Once per Year	2-3 Times per Month	Once per Week	2 Times per week	3-4 Times per Week	5-6 Times per Week	Every Day	1	2	3	4
Pizza or pizza pockets													

Eggs rolls, won tons, samosas, empanadas	○	○	○	○	○	○	○	○	○	○	○	○	○

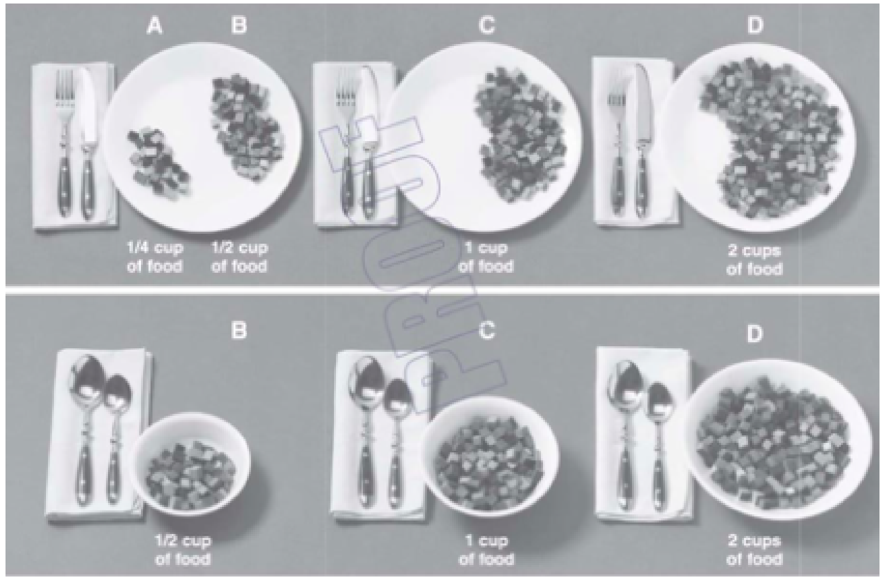

Q43. MEAT and CHICKEN

	How Often?									How much on those days? (see portion size pictures for A-B-C-D)			
	Never	A Few Times per 4 Months	Once per Year	2-3 Times per Month	Once per Week	2 Times per week	3-4 Times per Week	5-6 Times per Week	Every Day	A	B or medium piece	C	D or half chicken
Meat loaf, meatballs	○	○	○	○	○	○	○	○	○	○	○	○	○
Steak, roast beef, pot roast, including in frozen dinner or sandwiches	○	○	○	○	○	○	○	○	○	○	○	○	○
Tacos, burritos, enchiladas, tamales, tostadas, with meat or chicken	○	○	○	○	○	○	○	○	○	○	○	○	○
Ribs, spareribs	○	○	○	○	○	○	○	○	○	○	○	○	○
Pork chops, pork roast, cooked ham (including for breakfast)	○	○	○	○	○	○	○	○	○	○	○	○	○
Any other beef or pork dish like stew, pot pie, corned beef hash, child, Hamburger Helper, curry	○	○	○	○	○	○	○	○	○	○	○	○	○
	Never	A Few Times per 4 Months	Once per Year	2-3 Times per Month	Once per Week	2 Times per week	3-4 Times per Week	5-6 Times per Week	Every Day	A	B or medium piece	C	D or half chicken
Liver, including chicken livers or liverwurst	○	○	○	○	○	○	○	○	○	○	○	○	○
Pigs feet, neck bones, oxtails, tongue, chitlins	○	○	○	○	○	○	○	○	○	○	○	○	○
Veal, lamb, goat, deer meat, other game	○	○	○	○	○	○	○	○	○	○	○	○	○
Roasted or broiled chicken or turkey	○	○	○	○	○	○	○	○	○	○	○	○	○

Any other chicken or turkey dish, like chicken stew or curry, chicken salad, stir-fry, Chinese chicken dishes													

Q44. MEAT and CHICKEN

	How Often?									How many on those days?				
	Never	A Few Times per 4 Months	Once per Year	2-3 Times per Month	Once per Week	2 Times per week	3-4 Times per Week	5-6 Times per Week	Every Day	1 small	1 Large	2 pcs or 6 nuggets	3	4
Hamburgers, cheeseburgers, turkey burger, at home or from a restaurant														
Hot dogs or dinner sausage like Polish, Italian, chicken apple														
Slices of lunch meats like bologna, sliced ham, sliced turkey, salami														
	Never	A Few Times per 4 Months	Once per Year	2-3 Times per Month	Once per Week	2 Times per week	3-4 Times per Week	5-6 Times per Week	Every Day	1 small	1 Large	2 pcs or 6 nuggets	3	4
Fried chicken, including chicken fingers, chicken nuggets, wings, chicken patty														
Click to write Statement 5														

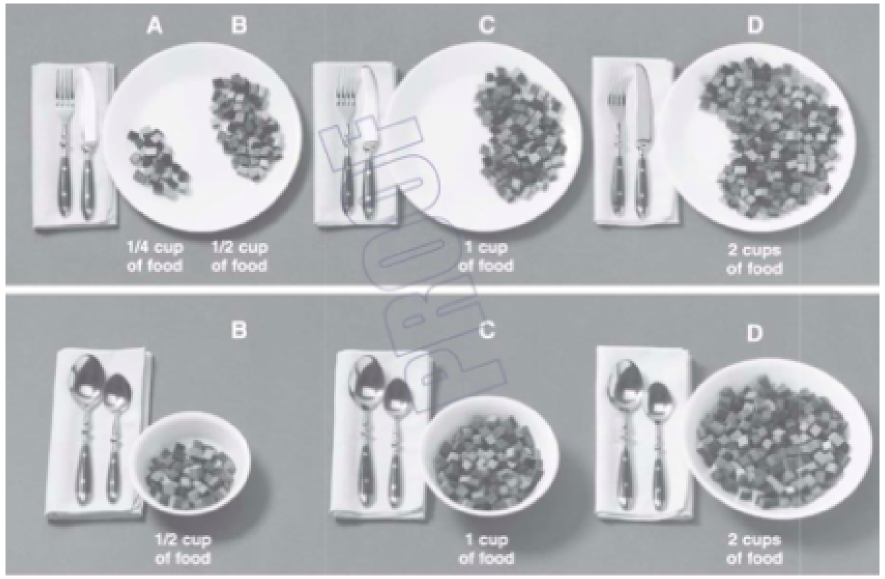

Q46. FISH, SEAFOOD

	How Often?									How much on those days? (see portion size pictures for A-B-C-D)			
	Never	A Few Times per 4 Months	Once per Year	2-3 Times per Month	Once per Week	2 Times per week	3-4 Times per Week	5-6 Times per Week	Every Day	A	B	C	D
Oysters													
Shellfish like shrimp, scallops, crab													
Tuna, tuna salad, tuna casserole													
	Never	A Few Times per 4 Months	Once per Year	2-3 Times per Month	Once per Week	2 Times per week	3-4 Times per Week	5-6 Times per Week	Every Day	A	B	C	D
Salmon, mackerel, sea bass, trout, sardines, herring, (without breading)													
Fried fish, fish sticks, fish sandwich, breaded fillets													
Any other fish													

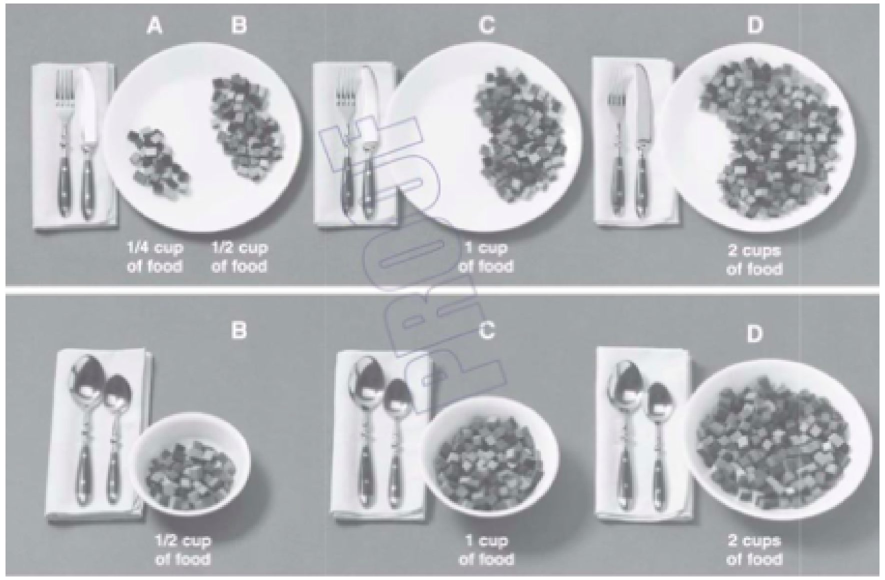

Q48. NUTS, SEEDS, SNACKS

	How Often?									How much on those days? (see portion size pictures for A-B-C-D)			
	Never	A Few Times per 4 Months	Once per Year	2-3 Times per Month	Once per Week	2 Times per week	3-4 Times per Week	5-6 Times per Week	Every Day	A	B	C	D
Peanuts, sunflower seeds, other nuts or seeds													
Whole grain crackers, like Wheat Thins, RyeKrisp, Ryvita, Wasa													

	Never	A Few Times per 4 Months	Once per Year	2-3 Times per Month	Once per Week	2 Times per week	3-4 Times per Week	5-6 Times per Week	Every Day	A	B	C	D
Any other cracker like saltines, Ritz, Cheese-Its, cheese-filled pretzels													
Tortilla chips or corn chips, like Fritos, Doritos, corn nuts													
Any other snack chips, like potato chips, Cheetos, Chex mix													

Q49. NUTS, SEEDS, SNACKS

	How Often?									How much on those days?				
	Never	A Few Times per 4 Months	Once per Year	2-3 Times per Month	Once per Week	2 Times per week	3-4 Times per Week	5-6 Times per Week	Every Day	1/2	1	2	3	4
Peanut butter or other nut butters (tbsps)														
Breakfast bars, cereal bars, granola bars (not energy or protein bars)														

Q50. NUTS, SEEDS, SNACKS

	How Often?									How much on those days?			
	Never	A Few Times per 4 Months	Once per Year	2-3 Times per Month	Once per Week	2 Times per week	3-4 Times per Week	5-6 Times per Week	Every Day	1 tbsp	2 tbsp	1/4 cup	1/2 cup
Walnuts or flax seeds (don't count flaxseed oil)													

Q51. NUTS, SEEDS, SNACKS

	How Often?									How much on those days?		
	Never	A Few Times per 4 Months	Once per Year	2-3 Times per Month	Once per Week	2 Times per week	3-4 Times per Week	5-6 Times per Week	Every Day	Small	Medium	Large
Energy or protein bars like Power Bar, Clif, Balance, Luna, South Beach, Atkins												

Q52. NUTS, SEEDS, SNACKS

	How Often?									How many cups on those days?			
	Never	A Few Times per 4 Months	Once per Year	2-3 Times per Month	Once per Week	2 Times per week	3-4 Times per Week	5-6 Times per Week	Every Day	1-2	3-6	7-9	10-12
Popcorn													

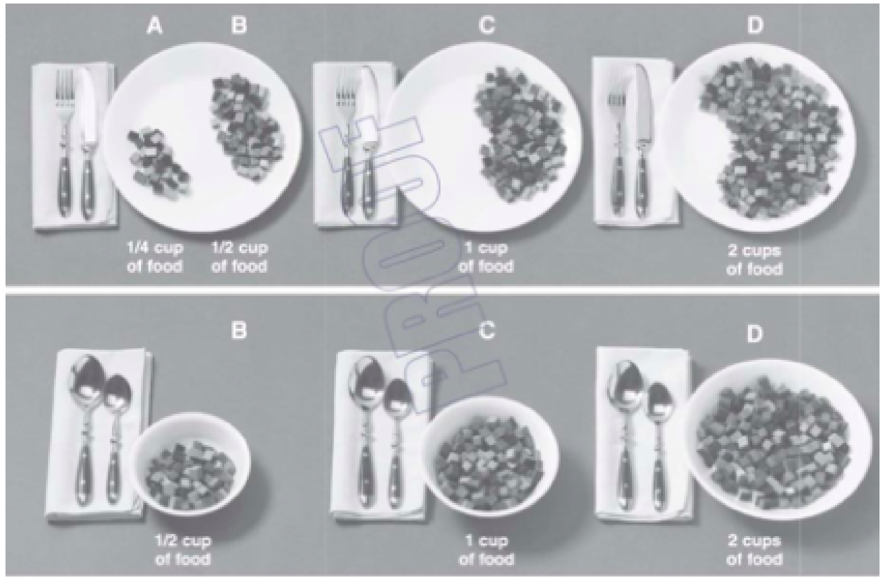

Q54. SWEETS and DESSERTS

	How Often?									How much on those days? (see portion size pictures for A-B-C-D)			
	Never	A Few Times per 4 Months	Once per Year	2-3 Times per Month	Once per Week	2 Times per week	3-4 Times per Week	5-6 Times per Week	Every Day	A	B	C	D
Ice cream, ice cream bars, frozen yogurt, fast food milkshakes													
Pudding, custard, rice pudding, flan													
Popsicles, jello. frozen fruit bars, slushies, sherbet (don't count sugar-free)													

Q55. SWEETS and DESSERTS

	How Often?									How many on those days?				
	Never	A Few Times per 4 Months	Once per Year	2-3 Times per Month	Once per Week	2 Times per week	3-4 Times per Week	5-6 Times per Week	Every Day	1/2	1	2	3	4+
Doughnuts														

	Never	A Few Times per 4 Months	Once per Year	2-3 Times per Month	Once per Week	2 Times per week	3-4 Times per Week	5-6 Times per Week	Every Day	1/2	1	2	3	4+
Cake or snack cakes like cupcakes, Twinkies, pound cake, banana bread														
Cookies or brownies														
Pumpkin pie, sweet potato pie														
Any other pie or cobbler, including fast food pies, snack pies														
Click to write Statement 4														

Q56. SWEETS and DESSERTS

	How Often?									How much on those days?		
	Never	A Few Times per 4 Months	Once per Year	2-3 Times per Month	Once per Week	2 Times per week	3-4 Times per Week	5-6 Times per Week	Every Day	1-2 tbsp	3-4 tbsp	1/2 cup
Chocolate or other flavoured sauces or syrup, on ice cream												

Q57. SWEETS and DESSERTS

	How Often?									How much on those days?			
	Never	A Few Times per 4 Months	Once per Year	2-3 Times per Month	Once per Week	2 Times per week	3-4 Times per Week	5-6 Times per Week	Every Day	Mini	Medium	Large	King
Chocolate candy, candy bars like Snickers, Hershey's, M&ms													

Q58. SWEETS and DESSERTS

	How Often?									How much on those days?			
	Never	A Few Times per 4 Months	Once per Year	2-3 Times per Month	Once per Week	2 Times per week	3-4 Times per Week	5-6 Times per Week	Every Day	1-2 pieces	1/2 package	1 package	2 packages
Any other candy, (NOT chocolate), like hard candy, Lifesavers, Skittles, Starburst, breath mints, chewing gum (NOT sugar free)													

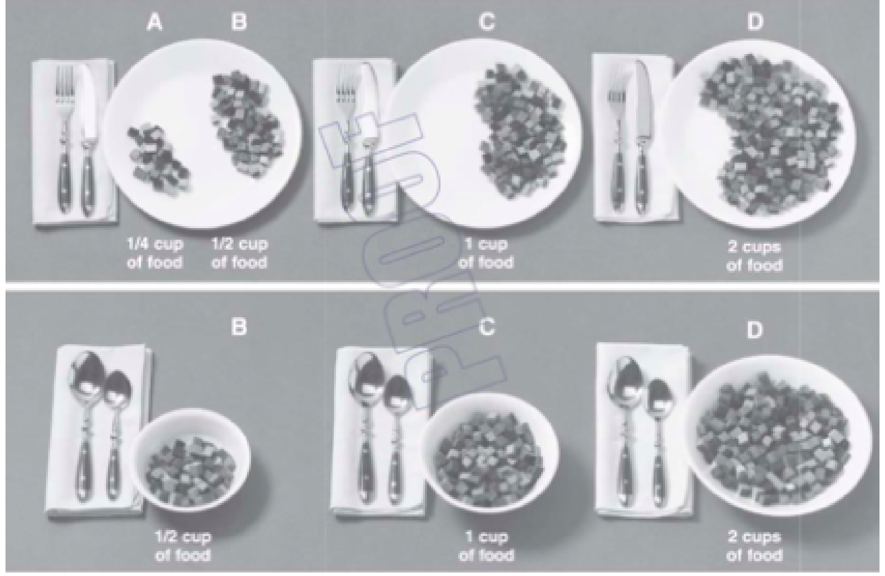

Q60. SPREADS, SAUCES, OTHER FOODS

Q61. SPREADS, SAUCES, OTHER FOODS

	How Often?									How much on those days? (see portion size pictures for A-B-C-D)			
	Never	A Few Times per 4 Months	Once per Year	2-3 Times per Month	Once per Week	2 Times per week	3-4 Times per Week	5-6 Times per Week	Every Day	A	B	C	D
Pickles, pickled vegetables, sauerkraut, kimchi	○	○	○	○	○	○	○	○	○	○	○	○	○

Q62. SPREADS, SAUCES, OTHER FOODS

	How Often?									How many tablespoons on those days?			
	Never	A Few Times per 4 Months	Once per Year	2-3 Times per Month	Once per Week	2 Times per week	3-4 Times per Week	5-6 Times per Week	Every Day	1	2	3	4
Margarine (NOT butter) on bread, rice, vegetables, or other foods	○	○	○	○	○	○	○	○	○	○	○	○	○
Butter (NOT margarine) on bread, rice, vegetables, or other foods	○	○	○	○	○	○	○	○	○	○	○	○	○
Click to write Statement 3	○	○	○	○	○	○	○	○	○	○	○	○	○
Click to write Statement 4	○	○	○	○	○	○	○	○	○	○	○	○	○

Q63. SPREADS, SAUCES, OTHER FOODS

	How Often?									How many teaspoons on those days?				
	Never	A Few Times per 4 Months	Once per Year	2-3 Times per Month	Once per Week	2 Times per week	3-4 Times per Week	5-6 Times per Week	Every Day	1/2	1	2	3	4
Mayonnaise, sandwich spreads														
Ketchup, salsa, chili sauce, chili peppers														
Mustard, barbecue sauce, soy sauce														
Jam, jelly, marmalade														

Q64. SPREADS, SAUCES, OTHER FOODS

	How Often?									How many cups on those days?		
	Never	A Few Times per 4 Months	Once per Year	2-3 Times per Month	Once per Week	2 Times per week	3-4 Times per Week	5-6 Times per Week	Every Day	1/4	1/2	1
Gravy, or other rich sauces like Alfredo, white sauce, mole, peanut sauce												

Q65. SPREADS, SAUCES, OTHER FOODS

	How Often?									How many shakes on those days?			
	Never	A Few Times per 4 Months	Once per Year	2-3 Times per Month	Once per Week	2 Times per week	3-4 Times per Week	5-6 Times per Week	Every Day	1-3	4-5	6-7	8+
Salt, added to your food at the table													

Q66. BEVERAGES

	How Often?								
	Never	A Few Times per 4 Months	Once per Year	2-3 Times per Month	Once per Week	2 Times per week	3-4 Times per Week	5-6 Times per Week	Every Day

	Never	A Few Times per 4 Months	Once per Year	2-3 Times per Month	Once per Week	2 Times per week	3-4 Times per Week	5-6 Times per Week	Every Day
Chocolate milk, cocoa, hot chocolate	○	○	○	○	○	○	○	○	○
Glasses of milk or soy milk (NOT counting on cereal, in coffee, or chocolate milk)	○	○	○	○	○	○	○	○	○
Meal replacement drinks like Slim Fast, Ensure, or high protein drinks or powders	○	○	○	○	○	○	○	○	○
Tomato juice, V-8, other vegetable juice	○	○	○	○	○	○	○	○	○
Real 100% orange juice or grapefruit juice (do NOT count orange soda or Sunny Delight)	○	○	○	○	○	○	○	○	○
Other 100% juices, like apple, grape, fruit blends, or fruit smoothies	○	○	○	○	○	○	○	○	○
Hi-C, cranberry juice cocktail, Hawaiian Punch, Tang	○	○	○	○	○	○	○	○	○
	Never	A Few Times per 4 Months	Once per Year	2-3 Times per Month	Once per Week	2 Times per week	3-4 Times per Week	5-6 Times per Week	Every Day
Drinks with some juice like Sunny Delight, Knudsen	○	○	○	○	○	○	○	○	○
Iced tea, homemade, instant or bottled, like Nestea, Lipton, Snapple, Tazo	○	○	○	○	○	○	○	○	○
Gatorade, Powerade, or other sports drinks	○	○	○	○	○	○	○	○	○
Energy drinks like Red Bull, Rockstar, Monster	○	○	○	○	○	○	○	○	○
Kool-Aid, lemonade, fruit flavoured drinks, like Crystal light, atole horchata, (NOT iced tea)	○	○	○	○	○	○	○	○	○
Soft drinks, soda, pop, like cola, 7-Up, orange soda, regular or diet	○	○	○	○	○	○	○	○	○
Beer or non-alcoholic beer	○	○	○	○	○	○	○	○	○
	Never	A Few Times per 4 Months	Once per Year	2-3 Times per Month	Once per Week	2 Times per week	3-4 Times per Week	5-6 Times per Week	Every Day
Wine or wine coolers	○	○	○	○	○	○	○	○	○
Liquor or mixed drinks, cocktails	○	○	○	○	○	○	○	○	○
Water, bottled or tap	○	○	○	○	○	○	○	○	○
Milky coffee drinks like latte, mocha, cappuccino, Frappucino	○	○	○	○	○	○	○	○	○
Coffee (brewed or instant), regular or decaf	○	○	○	○	○	○	○	○	○
Hot tea (NOT including herbal tea)	○	○	○	○	○	○	○	○	○

Q67. Beverages

How many 8 ounce servings on those days?

	1/2	1	2	3	4
Glasses of milk or soy milk, (NOT counting on cereal, in coffee, or chocolate milk)	○	○	○	○	○
Tomato juice, V-8, other vegetable juice	○	○	○	○	○
Real 100% orange juice or grapefruit juice (NOT orange soda or Sunny Delight)	○	○	○	○	○

Other 100% juices, like apple, grape, 100% fruit blends, or fruit smoothies	○	○	○	○	○

Q68. Beverages

	How many 12 ounce servings on those days?				
	1/2	1	2	3	4
Chocolate milk, cocoa, hot chcoclate	○	○	○	○	○
Hi-C, cranberry juice cocktail, Hawaiian Punch, Tang	○	○	○	○	○
Drinks with some juice like Sunny Delight, Knudsen	○	○	○	○	○

Q69. ON THOSE DAYS, how many cans or glasses of meal replacement drinks like Slim Fast, Ensure or high protein drinks or powders?

- ○ 1
- ○ 2
- ○ 3
- ○ 4

Q70. ON THOSE DAYS, how many 16 ounce glasses or bottles of iced tea (homemade, instant, bottled, like Nestea, Lipton, Snapple, Tazo) do you drink?

- ○ 1/2
- ○ 1
- ○ 2
- ○ 3

Q71. ON THOSE DAYS, how many ounces of sport drink (Powerade, Gatorade) do you drink?

- ○ One 16 ounce bottle
- ○ One 20 ounce bottle
- ○ Two 16 ounce bottles
- ○ Two 20 ounce bottles

Q72. ON THOSE DAYS, how much do you drink energy drinks (like Red Bull, Rockstar, Monster)?

- One 8 ounce can
- One 12-16 ounce can
- One 20 ounce can
- 24 ounces or more

Q73. ON THOSE DAYS, how much do you drink Kool-Aid, lemonade, fruit flavoured drinks like Crystal light?

- One 8 ounce can
- One 12-16 ounce can
- One 20 ounce can
- 30 ounces or more

Q74. ON THOSE DAYS - How much do you drink of soda, or pop like cola, 7-Up, orange soda, (regular or diet)?

- 1 can
- 20 ounce bottle
- 2 cans
- Big Gulp or 3 cans

Q75. ON THOSE DAYS - How much beer do you drink (including non-alcoholic beer)?

- 1 can
- 2 cans
- 3-4 cans
- 5+ cans or a large pitcher

Q76. ON THOSE DAYS - How much wine/wine coolers do you drink?

- 1/2 glass
- 1 glass
- 2 glasses, 1/2 bottle
- 4+ glasses

Q77. ON THOSE DAYS - How many drinks of liquor or mixed drinks like cocktails?

- 1
- 2
- 3
- 4+

Q78. ON THOSE DAYS - How many glasses of water do you drink?

- 1
- 2
- 3-4
- 5+

Q79. ON THOSE DAYS - How many ounces of milky coffee do you drink (like latte, mocha, cappuccino, Frappuccino)?

- 12 ounces
- 16 ounces
- 20 ounces
- 24+ ounces

Q80. ON THOSE DAYS - How many coffees (brewed or instant, regular or decaf) do you drink in a day?

- 1
- 2
- 3
- 4+

Q81. ON THOSE DAYS - How many cups of hot tea (not including herbal) do you drink in a day?

- 1
- 2
- 3
- 4+

Q82. The following questions are meant to specify types of food you consume.

Only select ONE unless instructed otherwise.

If you don't consume the food/drink in question select "Don't eat" or "Don't drink".

Q83. MILKY COFFEE DRINKS: What kinds do you usually drink?

- Frappuccino
- Cafe con leche

- Mocha
- Latte or cappuccino
- Some of each
- Dont drink

Q84. COFFEE: Is your coffee usually regular or decaf?

- Decaf
- Regular
- Both
- Don't drink

Q85. What are your milky coffee drinks usually made with?

- Whole milk
- 1 or 2% (reduced fat)
- Skim or non-fat
- Soy milk
- Something else
- Don't drink

Q86. What do you usually add to your coffee?

- Cream or half-n-half
- CoffeeMate, non dairy creamer
- Condensed milk
- Any other milk
- None of these

Q87. How many teaspoons of sugar (or honey) do you add to each cup of coffee?

0	1	2	3	4
○	○	○	○	○

Q88. HOT TEA: Is your hot tea usually regular or decaf?

- Decaf
- Regular
- Both
- Don't drink

Q89. What do you usually add to your hot tea?

- Cream or half-n-half
- CoffeeMate, non dairy creamer
- Condensed milk
- Any other milk
- None of these

Q90. How many teaspoons of sugar (or honey) do you add to each cup of tea?

0	1	2	3	4
○	○	○	○	○

Q91. MILK

- Whole milk
- Skim milk, non-fat
- 1% (low fat)
- 2% milk
- Soy milk
- Rice milk
- Almond milk, other
- Don't drink

Q92. SLIMFAST, ENSURE, OR HIGH PROTEIN DRINKS

- Slimfast, Ensure, regular
- Slimfast, Ensure, light or low-carb
- High protein drinks, regular
- High protein drinks, light or low-carb

- Don't drink

Q93. Real 100% orange or grapefruit juice

- Calcium-fortified
- Not calcium fortified
- Unsure
- Don't drink

Q94. Iced-tea

- Home-made, no sugar
- Home-made, with sugar
- Bottled, no sugar
- Bottled, pre-sweetened
- Don't drink

Q95. Energy drinks like Red Bull, Monster

Sugar-free	Regular	Don't drink
○	○	○

Q96. Drinks like Kool-Aid, lemonade, Crystal Light

Low-calorie, sugar-free	Regular	Don't drink
○	○	○

Q97. Soft drinks, soda, pop

Diet, low-calorie	Regular	Don't drink
○	○	○

Q98. Soft drinks, soda, pop

Has caffeine	No caffeine	Don't drink
○	○	○

Q99. Beer

- ○ Regular
- ○ Light
- ○ Non-alcoholic
- ○ Don't drink

Q100. Wine or wine cooler

- ○ Red wine
- ○ White wine
- ○ Both red and white wine
- ○ Don't drink

Q101. Cheese

Low-fat	Regular-fat	Don't eat
○	○	○

Q102. Yogurt

Plain (no sugar or fruit)	With fruit or other flavours
○	○

Q103. Yogurt

- ○ Low-fat
- ○ Non-fat
- ○ Regular (whole milk)
- ○ Don't eat

Q104. Salad dressing

- Low-fat,lite
- Fat free
- Regular
- Oil & vinegar
- Don't use

Q105. Spaghetti or lasagna

Meatless	With meat sauce or meatballs	Don't eat
○	○	○

Q106. Noodles, pasta

- Rarely whole grain
- Sometimes whole grain
- Usually whole grain
- Don't know/don't eat

Q107. Burgers

- Hamburger
- Cheeeseburger
- Turkey burger
- Don't eat

Q108. Beef or pork

- Avoid eating the fat
- Sometimes eat the fat
- Often eat the fat
- Don't eat

Q109. Chicken or turkey

- Avoid eating the skin
- Sometimes eat the skin
- Often eat the skin
- Don't eat

Q110. Hot dogs, dinner sausage

Beef or pork	Chicken or turkey, low-fat	Don't eat
◯	◯	◯

Q111. Lunch meats

Beef or pork	Chicken or turkey, low-fat	Don't eat
◯	◯	◯

Q112. Cakes, snack cakes, cupcakes

- Low-sugar, low-carb
- Low-fat
- Regular-fat
- Don't eat

Q113. Cookies, brownies

- Low-sugar, low-carb
- Low-fat
- Regular-fat
- Don't eat

Q114. Ice cream, frozen yogurt

- Low-sugar, low-carb
- Low-fat or frozen yogurt
- Regular
- Don't eat

Q115. Energy or protein bars

- High energy
- High protein
- Some of each
- Unsure
- Don't eat

Q116. Bagels, English muffins, rolls

- White
- Multi-grain
- 100% whole wheat
- Eat all kinds
- Don't eat

Q117. Burger, hot dog, submarine buns

- White
- Multi-grain
- 100% whole wheat
- Eat all kinds
- Don't eat

Q118. Bread

- White (not whole grain)
- 100% whole wheat

- Multi-grain, rye, other brown bread
- Eat some of each
- Don't eat

Q119. Tortillas

- Corn tortillas
- Flour tortillas (wheat)
- Eat all kinds or unsure
- Don't eat

Q120. Popcorn

- Air popped, fat free
- Low-fat or light
- Regular
- Caramel corn
- Don't know
- Don't eat

Q121. Crackers, pretzels

- Low-fat (including RyeKrisp, rice cakes, or plain pretzels)
- Regular-fat crackers or filled pretzels
- Don't know
- Don't eat

Q122. Mayonnaise or sandwich spreads

Low-fat, light	Regular	Don't eat
○	○	○

Q123. If you eat COLD cereals, what do you usually eat? Choose ONE or TWO that you eat the most often. If you usually eat just one kind, only choose one.

- All Bran Original
- Cinnamon Toast Crunch
- Grape Nuts
- Special K, plain

- All Bran Complete, Complete
- Apple Jacks, Cookie Crisp
- Bran Flakes
- Cap'n Crunch
- Cheerios, plain or Multi-Grain
- Cheerios, Honey Nut, flavours
- Chex, Wheat
- Chex, other
- Cocoa Krispies, Pebbles, Puffs
- Corn Flakes, Corn Puffs
- Corn Pops
- Fiber-One, Bran Buds
- Fruit Loops
- Frosted Flakes
- Frosted Mini-Wheats
- Granola
- Honey Bunches of Oats
- Kashi GOLEAN, Heart to Heart
- Life
- Lucky Charms, Fruity Pebbles
- Oatmeal Squares, Oat Bran
- Raisan Bran
- Rice Krispies, puffed rice
- Shredded Wheat
- Special K, flavours
- Total
- Wheaties
- OTHER sweet cereal
- OTHER unsweetened cereal
- OTHER whole grain cereal
- OTHER bran or fiber cereal
- Don't eat

Q124. Which fats or oils are used most often for cooking or frying (NOT baking) in your home? Mark only one or two

- Non-stick spray or none
- Butter or ghee
- Butter/margarine blend
- Stick margarine
- Soft tub margarine
- Low-fat margarine
- Olive oil
- Canola oil, safflower oil
- Corn oil, vegetable oil and blends
- Peanut oil
- Lard, fatback, or bacon fat
- Vegetable shortening, Crisco
- Other oil
- Don't know

Q125. Vitamins

	How Often?									For how many years?			
	Never	A Few Times per 4 Months	Once per Year	2-3 Times per Month	Once per Week	2 Times per week	3-4 Times per Week	5-6 Times per Week	Every Day	Less than 1 year	1-4 years	5-9 years	10+ years
Prenatal vitamins	○	○	○	○	○	○	○	○	○	○	○	○	○
Regular One-A-Day, Centrum, "senior vitamins or house brands of multi vitamins	○	○	○	○	○	○	○	○	○	○	○	○	○
Stress-tabs or B-Complex type	○	○	○	○	○	○	○	○	○	○	○	○	○
Antioxidant combination, eye formula	○	○	○	○	○	○	○	○	○	○	○	○	○
Vitamin A (not beta-carotene)	○	○	○	○	○	○	○	○	○	○	○	○	○
Vitamin B-6	○	○	○	○	○	○	○	○	○	○	○	○	○
Vitamin B-12	○	○	○	○	○	○	○	○	○	○	○	○	○
VitaminC	○	○	○	○	○	○	○	○	○	○	○	○	○

	Never	A Few Times per 4 Months	Once per Year	2-3 Times per Month	Once per Week	2 Times per week	3-4 Times per Week	5-6 Times per Week	Every Day	Less than 1 year	1-4 years	5-9 years	10+ years
Vitamin D	○	○	○	○	○	○	○	○	○	○	○	○	○
Vitamin E	○	○	○	○	○	○	○	○	○	○	○	○	○
Folic acid, folate	○	○	○	○	○	○	○	○	○	○	○	○	○
Calcium or antacids with calcium, like Tums	○	○	○	○	○	○	○	○	○	○	○	○	○
Iron	○	○	○	○	○	○	○	○	○	○	○	○	○
Zinc	○	○	○	○	○	○	○	○	○	○	○	○	○
Cod liver oil, other fish oils, Omega-3, flax seed oil, algae	○	○	○	○	○	○	○	○	○	○	○	○	○
Fiber supplements like Benefiber, Metamucil	○	○	○	○	○	○	○	○	○	○	○	○	○
	Never	A Few Times per 4 Months	Once per Year	2-3 Times per Month	Once per Week	2 Times per week	3-4 Times per Week	5-6 Times per Week	Every Day	Less than 1 year	1-4 years	5-9 years	10+ years

Q126. One-A-Day, Centrum, or other types of multi vitamins, do you usually take types that:

- ○ Contain minerals, iron, zinc, etc.
- ○ Do not contain minerals
- ○ Don't know
- ○ Don't take

Q127. How many milligrams of Vitamin C a day? (select closest amount)

- ○ 100
- ○ 250
- ○ 500
- ○ 750
- ○ 1000
- ○ 1500
- ○ 2000
- ○ 3000+
- ○ Don't take

Q128. How many IUs of Vitamin E a day? (select closest amount)

- ○ 100
- ○ 200
- ○ 300
- ○ 400
- ○ 600
- ○ 800
- ○ 1000
- ○ 2000+
- ○ Don't take

Q129. How many milligrams of calcium a day? (select closest amount)

- 100
- 350
- 650
- 1250+
- Don't take

Q130. How many IUs of Vitamin D a day?

- 400
- 600
- 800
- 1000
- 2000
- 3000
- 4000
- 5000+
- Don't take

Q131. Type of omega-3 supplements (mark all that apply)

- Fish oil
- Flax oil, hemp oil, other seed oil
- Krill oil
- Algae
- Don't know
- Don't take

Q132. SOME LAST QUESTIONS ABOUT YOU

Q133. About how many servings of vegetables do you eat, not counting salad or potatoes? (1 serving = 1/2 cup).

- Rarely
- 1-2 per week
- 3-4 per week
- 5-6 per week
- 1 per day
- 1 1/2 per day
- 2 per day
- 3 per day
- 4+ per day

Q134. About how many servings of fruit do you eat, not counting juices? (1 serving = 1/2 cup).

- Rarely
- 1-2 per week
- 3-4 per week
- 5-6 per week
- 1 per day
- 1 1/2 per day
- 2 per day
- 3 per day
- 4+ per day

Q135. How often do you eat foods prepared at home theare cooked or fried in fat or oil?

- Rarely
- 1-2 per week
- 3-4 per week
- 5-6 per week
- 1 per day
- 1 1/2 per day
- 2 per day
- 3 per day
- 4+ per day

Q136. During a regular day, how many meals and snack do you usually eat?

Meals per day ▼

Snacks per day ▼

Location Data

Location: (43.114196777344, -79.203498840332)

Source: GeoIP Estimation

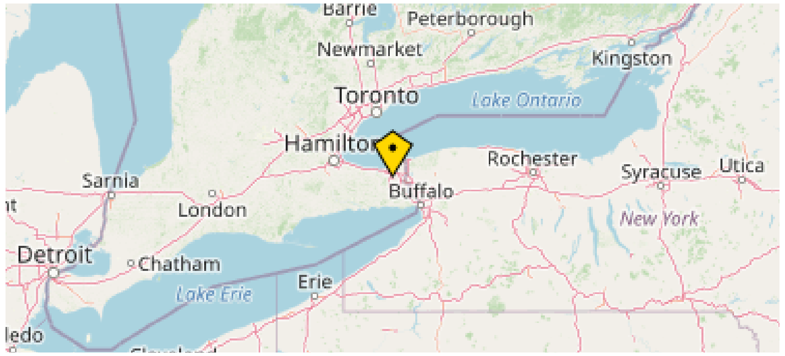

Supplement: Supplementary file 1 [file biology-12-00326-s001.zip › biology-2188589-supplementary.pdf]
